# Genetic Implications of Plant Sourcing Strategies for Salt Marsh Restoration

**DOI:** 10.1111/eva.70292

**Published:** 2026-07-29

**Authors:** A. Randall Hughes, Serena Caplins, Neelima Cherukuri, Madeleine DuChaussee, Gabriela Garcia, Peter Garneau, Tessa McClain, Andrew McQueen, Katherine Sperry, Sarit Truskey, Erik E. Sotka

**Affiliations:** ^1^ Marine Science Center Northeastern University Nahant Massachusetts USA; ^2^ Research Computing Northeastern University Boston Massachusetts USA; ^3^ US Army Corps of Engineers Engineer Research and Development Center Vicksburg Mississippi USA; ^4^ Department of Biology College of Charleston Charleston South Carolina USA

**Keywords:** conservation genetics, population genetics, restoration, restriction site‐associated DNA sequencing, salt marsh, single nucleotide polymorphism (SNP), *Spartina alterniflora*, *Spartina patens*

## Abstract

Ecosystem restoration aims to recreate the ecological structure, processes, and function of a reference native ecosystem. The structure and composition of restored ecosystems often differ from natural ecosystems, at least in part due to human decisions about which ecosystem characteristics and functions to prioritize. We generally lack an understanding of the precise steps in the restoration process that contribute to those differences, but decisions regarding how and where to source biological material are likely to play a role. Here, we use salt marshes as a system to test the potential for sourcing decisions to influence the genetic diversity and composition of two dominant plant species, *Spartina alterniflora* and *S. patens* , in restored salt marshes. We show that there is weak but detectable genetic differentiation across local populations that could serve as sources for restoration efforts, though the magnitude of this differentiation varied by analysis and across the two plant species. In addition, the degree of differentiation is generally greater among plant stocks produced from seed from these potential source populations than among the original populations themselves. Most clearly, plants from commercial nursery sources generally have higher genetic diversity and were highly differentiated from both the local populations and seedlings produced from seed from those local populations. These results are consistent with recent studies showing that restored marshes have distinct genetic characteristics from adjacent natural marshes. Given the demonstrated links between plant genetic variation and ecosystem function and resilience, differences in genetic variation resulting from sourcing decisions have the potential to impact the success of restoration efforts and should be an explicit component of restoration practice.

## 1 Introduction

Ecosystem restoration can be characterized as the intentional intervention in an ecosystem that has been degraded, with the aim of recreating the ecological structure, processes, and function of a reference native ecosystem (Holl et al. 2022). Thus, the structure and composition of restored ecosystems are largely a consequence of human decisions about which ecosystem characteristics and functions to prioritize. For example, many restoration projects prioritize the cover and abundance of native species, leading to restoration practices that favor widespread and readily available species with high survival, thus promoting biotic homogenization (Holl et al. 2022). In addition, restored populations often have reduced genetic diversity, distinct genetic composition, and/or lower diversity of functional traits than natural systems, which may compromise their persistence and/or resilience (Espeland et al. 2017; Holl et al. 2022). As a result, restored ecosystems are often ecologically and evolutionarily distinct compared to natural ecosystems (Espeland et al. 2017; Höfner et al. 2022; Holl et al. 2022). Yet, we often lack an understanding of the precise steps in the restoration process that contribute to those differences, hindering our ability to achieve a different outcome.

In many restoration projects, natural colonization is not sufficient to achieve restoration goals, and practitioners must decide how to source organisms that can be actively added to the system (Espeland et al. 2017; Jones et al. 2018). Identifying appropriate sources of native species is critical to these efforts, and there are two primary strategies for doing so: (1) collecting from wild populations or (2) obtaining from commercial sources. Because wild sources are finite and in many cases declining, commercial sources, when they exist, are often the preferred sourcing strategy. We typically know little about the genetic diversity or composition of commercial sources, and in some cases, they have been shown not to meet the demands for diverse, genetically appropriate material (Andres et al. 2024; Goodale et al. 2023; Pedrini et al. 2023). These limitations have led to increasing calls for developing decentralized and localized capacity for sustainable production of material for scaling up restoration efforts (Goodale et al. 2023), though the genetic impacts of localized production are also largely uncharacterized. At a minimum, such efforts would benefit from an understanding of the degree of genetic variation within and among local populations, as well as how that variation may shift during the production process (e.g., via differential germination under greenhouse conditions; Espeland et al. 2017). Overall, examining the diversity and composition of potential source stocks is needed to inform practitioner decision‐making regarding the likely genetic implications of different restoration practices.

We examined the genetic implications of restoration sourcing strategies in two widespread salt marsh plant species, *Spartina alterniflora* and *Spartina patens* , that form the foundation of extensive salt marsh ecosystems along the Atlantic and Gulf coasts of the United States (U.S.). Salt marshes provide essential ecosystem services which are increasingly valued for their contributions to coastal resiliency and protection of coastal infrastructure (Pannozzo et al. 2021; Zu Ermgassen et al. 2021; Liu et al. 2024). These ecosystems are under threat globally due to anthropogenic and natural pressures (Campbell et al. 2022; Alemu et al. 2024). Therefore, there are significant resources aimed at restoring degraded salt marsh habitats to regain these critical functions and values, and they are the most widely restored coastal ecosystem in the U.S. (Grabowski et al. 2012). In systems dominated by habitat‐forming plant species, practitioner decision‐making regarding restoration and sourcing approach is likely to have a direct influence on the genetic composition and diversity of the restored population (Kettenring et al. 2014; Espeland et al. 2017), as has been shown in marshes in the northeast U.S. (Crosby et al. 2024; Sperry et al. 2025). Further, genetic and phenotypic variation in these marsh plants impacts the ecology and function of the system (Hughes 2014; Zerebecki et al. 2017, 2021; Noto and Hughes 2020; Zerebecki and Hughes 2023). *Spartina alterniflora* and *S. patens* can both be propagated from seed (Zerebecki et al. 2021, Bartholet et al. 2024), and thus source material for active planting can be produced from seed from wild marshes or purchased from commercial nursery stocks, allowing for a comparison of the genetic consequences of these different sourcing decisions.

**FIGURE 1:**
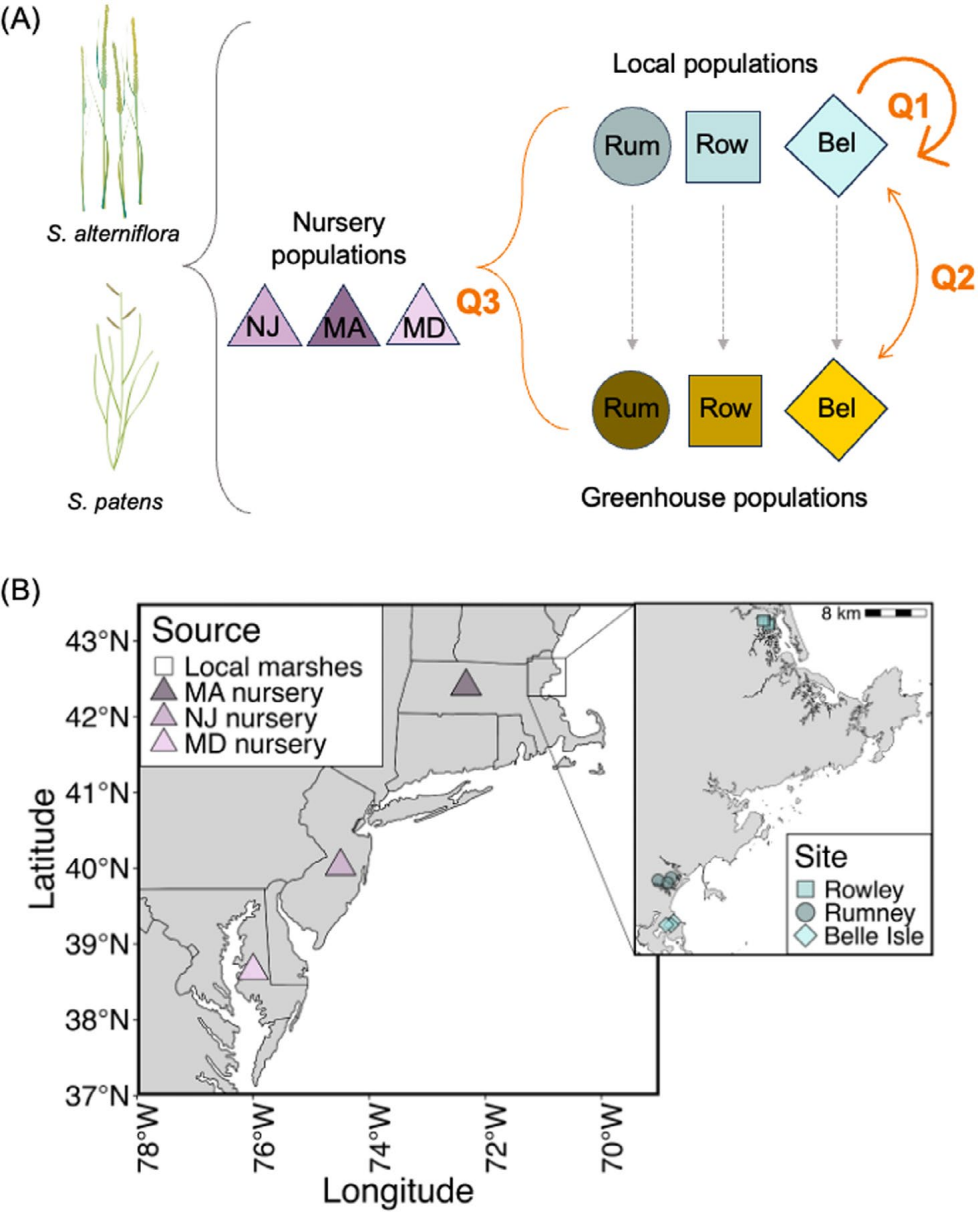
(A) Conceptual diagram of the study design. We tested the genetic implications of restoration sourcing strategies in two widespread salt marsh species, *Spartina alterniflora* and *Spartina patens* . We sequenced single nucleotide polymorphisms (SNPs) across multiple nursery populations, local wild populations, and greenhouse populations produced from those local populations to examine three questions: (Q1) Are there differences in genetic diversity or composition across local marshes? (Q2) Does genetic diversity or composition differ between local marshes and the greenhouse populations produced from seed collected at those sites? (Q3) Do plants from commercial nursery sources differ in genetic composition or diversity from local or greenhouse populations? (B) Map of the nursery and local marsh populations (inset). Nursery populations (triangles) came from New Jersey (NJ), Massachusetts (MA), and Maryland (MD) and local marsh populations and greenhouse populations came from Rumney Marsh Reservation (Rum; circles), the Great Marsh in Rowley (Row; squares), and Belle Isle Marsh Reservation (Bel; diamonds), all in Massachusetts (inset).

In this study, we used restriction‐site associated sequencing (RADseq) to genotype single nucleotide polymorphisms (SNPs) and address the following questions across multiple populations of *S. alterniflora* and *S. patens* (Figure 1): (1) Are there significant differences in genetic diversity or composition across different wild populations near the restoration site (“local”)? (2) Does genetic diversity or composition change during the greenhouse germination and production process in the resulting plants produced from seed in the greenhouse (“greenhouse”) relative to their local wild counterpart? (3) Do plants from commercial nursery sources (“nursery”) differ in composition or diversity from local and/or greenhouse populations produced from seed? We leveraged interviews with multiple commercial nurseries (Kollars and Hughes, unpublished data) to inform our seed collection, germination, and propagation methods so as to represent common practices. We hypothesized that local wild populations would exhibit moderate genetic diversity and slight genetic differentiation. In addition, we expected that genetic diversity would be lower and differentiation higher in the greenhouse plant stocks produced from seed relative to their local source populations due to genetic drift and/or selection during the propagation process (Espeland et al. 2017). Further, we hypothesized that plant stocks from nursery sources would have higher diversity and greater genetic differentiation from the local and greenhouse populations (Crosby et al. 2024). We calculated pairwise relatedness and heterozygosity as metrics of genetic diversity, and we conducted principal components analysis (PCA), calculated pairwise F_ST_, and ran an Analysis of Molecular Variance (AMOVA) to evaluate population structure and genetic differentiation.

## 2 Materials and Methods

### 2.1 Sampling Local Marsh Populations

In September 2022, we collected leaf tissue samples of *S. alterniflora* and *S. patens* from three subsites at each of three wild (i.e., unrestored) marshes in Massachusetts: Belle Isle Marsh Reservation (Bel), Rumney Marsh Reservation (Rum), and the Great Marsh in Rowley (Row; *N* = 9 subsites; Table 1, Figure 1). These marshes were selected due to their potential as local sources for regional restoration efforts planned for East Boston in support of broader coastal storm risk management goals being investigated by the City of Boston, by the U.S. Army Corps of Engineers (USACE), and by the USACE Engineering with Nature program (City of Boston 2022; McQueen et al. 2025). At each site, we collected a leaf sample from an individual plant every 2–3 m along one 40‐m or two 20‐m transects within the lower elevations of the marsh where *S. alterniflora* is taller (i.e., the “tall zone”), and within the adjacent higher elevation zone of *S. patens* , for a total of 20 samples per subsite. Samples were preserved with silica beads at the lab.

**TABLE 1:** Information for the local wild marshes from which we collected leaf tissue samples and reared greenhouse plants from seed. Local population *N* and Greenhouse population *N* refer to the number of individuals included in the genetic analyses.

Species	Site	Subsite	Latitude	Longitude	Date of seed collection	Local population *N*	Greenhouse population *N*
*S. alterniflora*	Belle Isle	L‐Berm	42.387	70.990	Sept 14 2022	20	0
	Belle Isle	Rosie's Pond	42.387	70.998	Sept 14 2022	20	20
	Belle Isle	Key	42.391	70.986	Sept 14 2022	20	0
	Rumney	Far	42.437	70.996	Sept 15 2022	20	20
	Rumney	Hamilton	42.443	70.990	Sept 15 2022	20	20
	Rumney	Terrace	42.440	71.011	Sept 15 2022	20	20
	Rowley	Stackyard	42.745	70.832	Sept 27 2022	20	50
	Rowley	Law's	42.748	70.843	Sept 27 2022	20	0
	Rowley	Patmos	42.748	70.840	Sept 27 2022	20	20
*S. patens*	Belle Isle	L‐Berm	42.388	70.991	Sept 14 2022	20	0
	Belle Isle	Rosie's Pond	42.388	70.999	Sept 14 2022	20	0
	Belle Isle	Key	42.391	70.986	Sept 14 2022	20	20
	Rumney	Far	42.437	70.995	Sept 15 2022	20	14
	Rumney	Hamilton	42.443	70.991	Sept 15 2022	20	12
	Rumney	Terrace	42.440	71.011	Sept 15 2022	20	13
	Rowley	Stackyard	42.745	70.832	Sept 27 2022	20	50
	Rowley	Stackyard South	42.743	70.834	Sept 27 2022	20	50
	Rowley	Patmos	42.746	70.840	Sept 27 2022	20	20

### 2.2 Producing Stocks From Seed From Local Marsh Populations

Concurrent with leaf tissue sampling, we collected seed‐bearing flowers of *S. alterniflora* and *S. patens* in the same areas of each of the same sites. Given differences in seed numbers per flowering stem across species, we collected ~75 *S. alterniflora* flowering stems and ~150 *S. patens* flowering stems per subsite. All plants from which flowers were collected were separated by at least 0.5 m. Flowering stems were placed in plastic bags separated by species and subsite.

We transported seeds to our greenhouse, where we removed seeds from stems and dried them in open plastic bags separated by species and subsite for 5–7 days. We then combined all seeds from a given subsite into aluminum trays (~23 × 33 cm). For *S. alterniflora* , we added approximately 2 L of 16 ppt brine water made with commercial salt (Instant Ocean, Blacksburg, Virginia) to fully cover the seeds and covered them with the lid. After another 10 days in the greenhouse, we replenished the trays with freshly made brine water and moved them to the refrigerator for cold storage. For *S. patens* , we held seeds dry at ambient temperature and sunlight for an additional 10 days before moving trays to cold (4°C ± 1°C) and dark storage in a refrigerator. Based on further conversations with commercial growers and further review of the literature, we temporarily removed *S. patens* trays from cold storage 60 days post‐collection and added enough 16 ppt brine water to fully cover the seeds.

In late January 2023, we moved all seed trays to the greenhouse at ambient light and temperature. We checked the trays for seeds that were beginning to germinate at least twice weekly until April 2023. Germinating seeds were counted and planted in 38‐cell seedling flats filled with commercial seedling starter soil (Coast of Maine Sprout Island blend) in the greenhouse. They were exposed to ambient light and temperature, irrigated with freshwater daily for 45 min, and flooded twice weekly with seawater.

In September 2023, we censused the number of seedlings of each species per subsite in our greenhouse stock. Three *S. alterniflora* subsites and two *S. patens* subsites had fewer than 5 seedlings surviving, so they were not considered further (Table 1). Three additional *S. patens* subsites had between 10 and 20 seedlings surviving, so we collected leaf tissue from each of those survivors (Table 1). One *S. alterniflora* subsite and two *S. patens* subsites had over 200 seedlings; we randomly selected 50 of each to collect leaf tissue. We collected leaf tissue from 20 seedlings from all remaining subsites (Table 1). Tissue was preserved on silica beads until extraction.

### 2.3 Sampling Commercial Nursery Stocks

In August 2023, we ordered 150 “plugs” of *S. alterniflora* and *S. patens* from three commercial nurseries along the Atlantic coast of the U.S. (from Maryland (MD), New Jersey (NJ), and Massachusetts (MA)) that have been proposed as sources for regional restoration efforts. The location of the marshes that served as sources for the nursery stocks was not provided. Nursery plugs were held in the greenhouse under ambient conditions. In September, concurrent with the sampling of the local seedling stock, we collected leaf tissue from 15 randomly selected seedlings of each species per nursery. Tissue was preserved on silica beads until extraction.

### 2.4 DNA Extraction and Sequencing

Tissue samples were processed in October 2023. DNA purification and extraction followed methods described in Hughes and Lotterhos (2014). In brief, approximately 0.25 g of tissue was ground in a mill grinder (RETSCH MM 400), and DNA was extracted and purified using Omega Bio‐Tek E‐Z Plant DNA kits. Double digest restriction‐site‐associated DNA (ddRAD) libraries with individually barcoded samples were prepared in separate batches for the two *Spartina* species following protocols in Parchman et al. (2012). We digested genomic DNA with two restriction enzymes, *EcoRI* and *MseI*, and ligated adaptors containing unique 8–10 bp barcodes to the digested DNA of each individual. The products were then PCR amplified in two independent reactions with standard Illumina primers. All amplicons were pooled and shipped to the University of Texas at Austin Genomic Sequencing and Analysis Facility (UT Austin GSAF; Austin, TX), which used Pippin Prep to isolate the 300–450 bp fraction. For the two batches, we randomly assigned samples in groups of 2–4 from collected subsites to plate positions and checked for sufficient interspersion to minimize batch effects associated with library preparation procedures. Both batches were sequenced individually with 100‐base pair single‐end sequencing on two lanes (full run) on the Illumina NovaSeq 6000 platform at the University of Texas at Austin Genomic Sequencing and Analysis Facility (Austin, TX).

### 2.5 Mapping and SNP Calling

We mapped reads to the *S. alterniflora* reference genome (GWHCBIM00000000; GenBank Acccession: JARYIK000000000, Chen et al. 2024) and hard called SNPs in ipyrad (v0.9.95; Eaton and Overcast 2020). In ipyrad, we set max_barcode_mismatch to 0, and filter_adapters equal to 2 for strict adapter filtering, the maxdepth to 4000, and set the max_alleles_consens to 2 for diploid SNPs. We used the bam files generated by ipyrad to generate genotype likelihoods in angsd (v1.16; Korneliussen et al. 2014) using the GATK model (−gl 2). For both hard‐called SNPs and genotype likelihood values, we removed SNPs with a minor allele frequency less than 0.05. We further filtered SNPs by genotype quality (max_low_qual_bases (*Q* < 20) = 5) and for missingness across sites, retaining sites that were shared across 80% of samples with vcftools (v0.1.17). We also filtered the genotype likelihood SNPset for linkage disequilibrium and pruned sites suspected of linkage using the programs ngsLD (v1.2.1) and prune_graph (v0.4.0), respectively. For the hard called SNPs, we filtered by a minimum distance of 5000 base pairs using vcftools (v0.1.17). We provided histograms of minor allele frequency, observed vs. expected heterozygosity, and missing data per locus per individual in the supplement (Figures S1–S3).

### 2.6 Relatedness and Heterozygosity

We calculated pairwise relatedness (rab) based on genotype likelihoods in ngsRelate (v2; Korneliussen and Moltke 2015) and estimated observed heterozygosity on hard‐called SNPs in ipyrad (v0.9.95). Both metrics were calculated by subpopulation for both *S. alterniflora* and *S. patens* . We ran separate Analyses of Variance (ANOVAs) to test whether relatedness or heterozygosity varied by source type (nursery, local, greenhouse) for each species. When source type was significant, and the assumptions of ANOVA satisfied (normality of residuals, homogeneity of variances, and independence of observations) we used Tukey's post hoc tests with alpha = 0.05 to test for significant differences among all pairwise comparisons.

### 2.7 Population Structure

We used genotype likelihoods as input to PCAngsd (v1.21; Meisner and Albrechtsen 2018) to calculate principal covariance matrices. We grouped samples for both species by: all samples, local, greenhouse, local + greenhouse, and nursery. We used likelihoods as input to NGSadmix (v1.21; Skotte et al. 2013) to perform estimates of population assignment and admixture for clusters (k) 2–5, and *maxiter* (iterations) set to 5000. We visualized NGSadmix structure plots in R using the pophelper package (Francis 2017). Pairwise *F*_ST_ was estimated in R with the package heirfstat using the hard‐called SNPs from each species (v0.5‐11; Goudet 2005). We performed Analyses of Molecular Variance (AMOVA) with an imputed dataset of genotype calls. We then imputed missing calls with the genotype that was most common across the entire dataset and calculated Nei's D between pairwise samples using (StAMPP; Pembleton et al. 2013) and then AMOVA using pegas (Paradis 2010).

## 3 Results

### 3.1 Mapping and SNP Calling

We obtained an average of 44.41 MB of raw sequence data for *S. alterniflora* and 44.39 for *S. patens* . We found 51.78% of *S. alterniflora* reads and 20.66% of *S. patens* reads mapped to the *S. alterniflora* reference genome. After filtering for missing data, quality, depth, minor allele frequency, and LD (linkage disequilibrium) or distance, we obtained 4213 hard called SNPs and 7008 genotype likelihoods across 47 contigs and 328 individuals for *S. alterniflora*, and 582 hard called SNPs and 5695 genotype likelihoods across 42 contigs and 350 individuals for *S. patens* . We removed one individual from each of 11 potential clonal pairs of *S. alterniflora* identified in the relatedness analysis (6 removals total; see next paragraph; Table S1), leaving us with 322 individual samples of *S. alterniflora* and 350 individual samples of *S. patens* .

### 3.2 Relatedness and Heterozygosity

**FIGURE 2:**
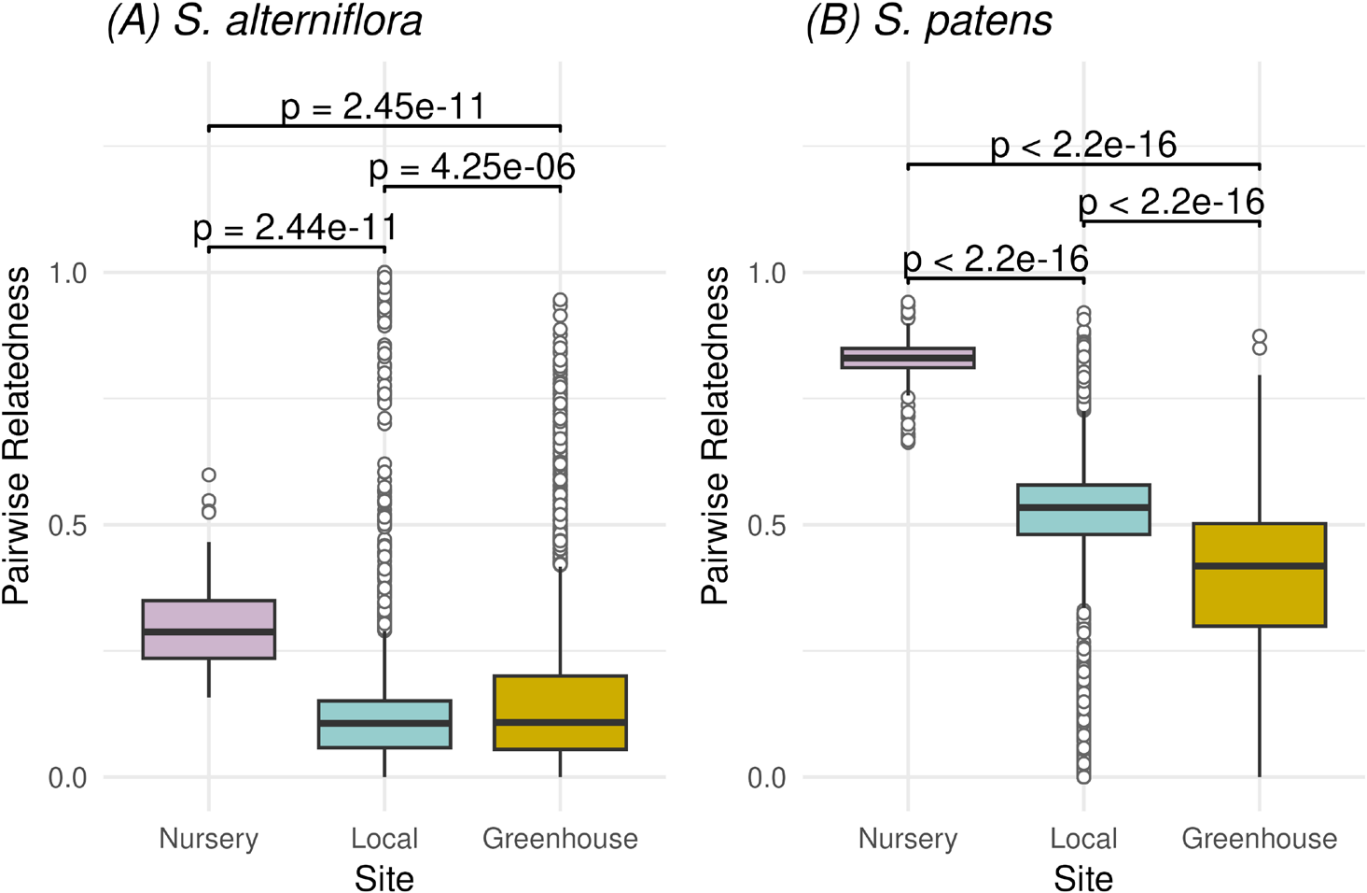
Box plots of pairwise relatedness between individuals collected from local wild marshes (Local; blue), produced from seed from those local marshes (Greenhouse; yellow), and purchased from commercial nurseries (Nursery; pink) for (A) *S. alterniflora* and (B) *S. patens* .

We found the average pairwise relatedness across all sources to be 0.072 (SD ±0.105) for *S. alternifora* and 0.345 (SD ±0.223) for *S. patens* (Figure 2). We found relatedness values of 0.999 for 11 out of 53,301 analyzed pairs in *S. alterniflora*, suggestive of clones. Suspected clones were predominantly from samples from the same site, and in one case were from greenhouse and local samples (Table S1). We also found 3 individuals with low heterozygosity that occurred in more than one of the 11 pairs. We omitted these 3 individuals, and we randomly selected one individual from each of the remaining suspected clonal pairs to omit from further analysis .

Relatedness differed significantly across local, greenhouse, and nursery samples of *S. alterniflora* (*F*_2,8009_ = 70.84, *p* < 0.001; Figure 2A, Table S2). Posthoc comparisons indicated that nursery samples (average rab ~0.3) had higher relatedness than both local (rab ~0.1) and greenhouse samples (rab ~0.1; TukeyHSD *p* < 0.001), and local samples on average had higher relatedness than greenhouse samples (TukeyHSD *p* < 0.001), with the exception of greenhouse samples from Belle Isle and Rowley, which had higher relatedness than local samples (Figure S4; Table S3).

For *S. patens* , we did not find pairwise relatedness values approximating 1, and only 3 pairs had a pairwise relatedness of 0.94. As with *S. alterniflora* , relatedness differed significantly across local, greenhouse, and nursery samples of *S. patens* (*F*_2,9675_ = 1526, *p* < 0.001; Figure 2B; Table S4), with nursery samples having higher pairwise relatedness than both local and greenhouse samples (TukeyHSD *p* < 0.001), and local samples typically having higher relatedness than greenhouse samples (TukeyHSD *p* < 0.001), both overall and when comparing local and greenhouse samples from the same site (Figure S4B, Table S5). There were also some differences in relatedness among particular subpopulations for both species (Figure S4B). For example, the Rumney local population had higher relatedness than the Rowley and Belle local populations in *S. patens* , and the MD nursery had higher relatedness than the MA and NJ nurseries (Figure S4B, Table S5) in both species.

**FIGURE 3:**
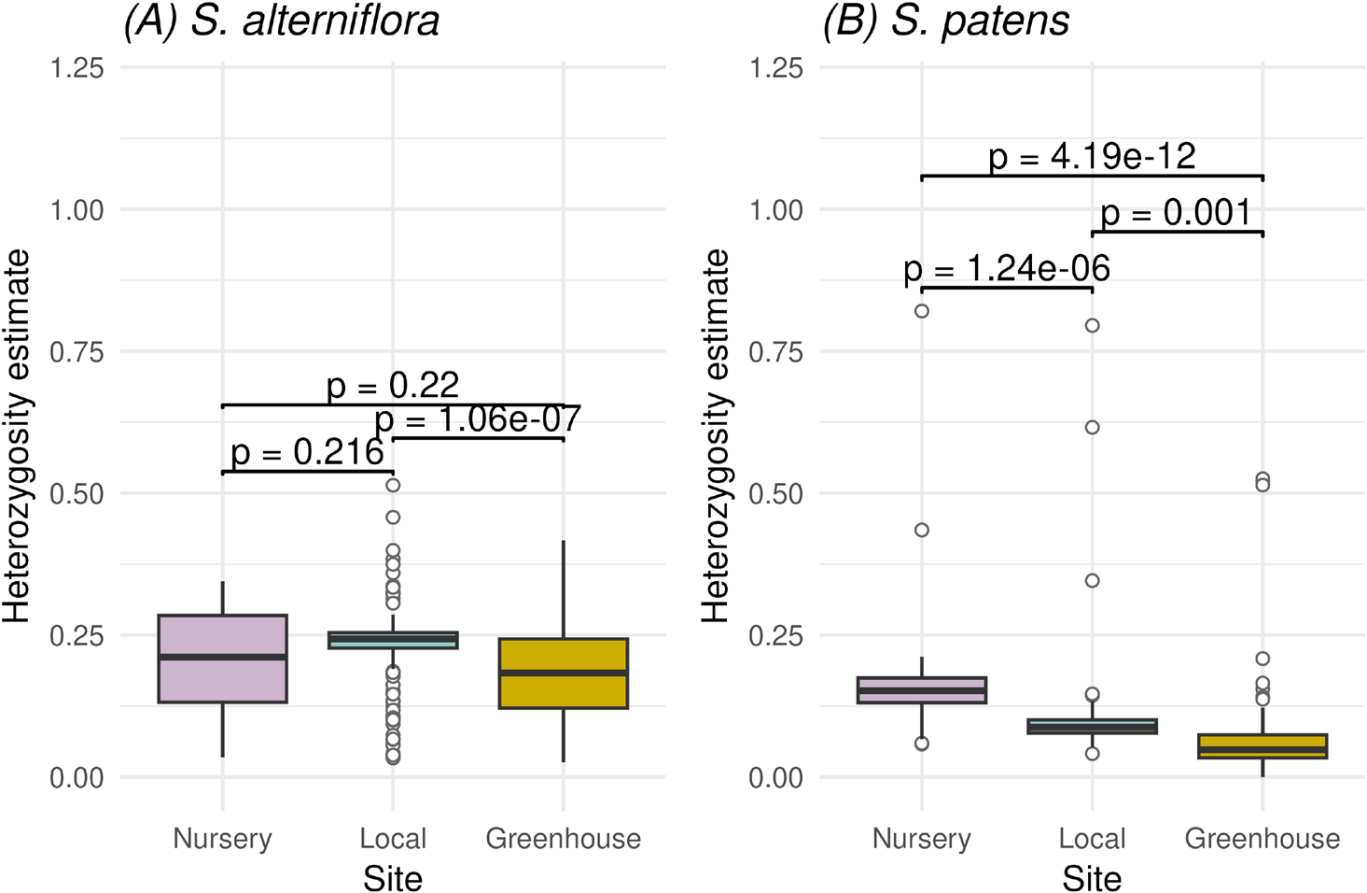
Box plots of observed heterozygosity between individuals collected from local wild marshes (Local; blue), produced from seed from those local marshes (Greenhouse; yellow), and purchased from commercial nurseries (Nursery; pink) for (A) *S. alterniflora* and (B) *S. patens* .

With clones removed, heterozygosity was estimated to be an average of 0.198 with a standard deviation of 0.085 for *S. alterniflora* , and an average of 0.089 with a standard deviation of 0.085 for *S. patens* . For both *S. alterniflora* and *S. patens* , heterozygosity differed across local, greenhouse, and nursery samples (*F* > 15.00, *p* < 0.001; Figure 3). Post hoc comparisons indicated that for both species, greenhouse samples had significantly lower heterozygosity than local samples ( *S. alterniflora* TukeyHSD *p* < 0.001; *S. patens* TukeyHSD *p* = 0.001). In addition, for *S. patens* both the local and greenhouse samples had significantly lower heterozygosity than the nursery populations (TukeyHSD *p* < 0.001 for both comparisons). Neither local and nursery samples nor nursery and greenhouse samples differed in heterozygosity for *S. alterniflora* (TukeyHSD *p* > 0.20). There were also differences across sites for both species. For *S. alterniflora* , the Rumney local population had higher heterozygosity than the MA nursery, and both the Rumney and Belle Isle local populations had higher heterozygosity than the Rumney and Rowley greenhouse populations (Figure S5A, Table S6). There were considerably more differences in heterozygosity across sites for *S. patens* (Figure S5B, Table S7); in general, these patterns mimicked the differences across source types, with nurseries having the highest heterozygosity, followed by the local populations, followed by the greenhouse sources.

### 3.3 Population Structure

**FIGURE 4:**
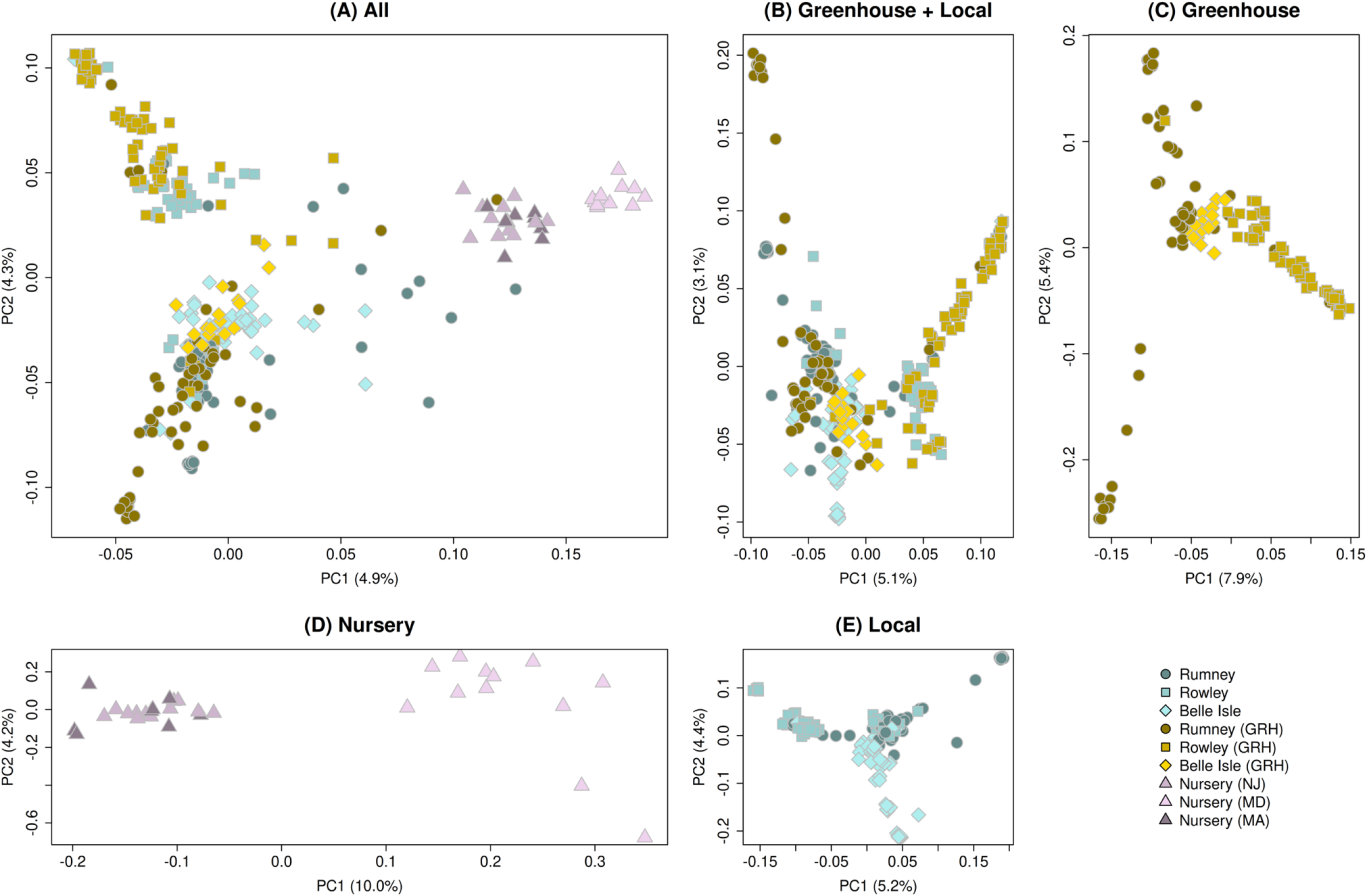
Principal component analysis for *S. alterniflora* for (A) all samples, (B) local marsh samples and the corresponding greenhouse seedlings, (C) greenhouse seedlings only, (D) nursery samples only, and (E) local marshes only. Shades of blue indicate samples from local marshes; shades of gold indicate seedlings produced in the greenhouse from seed from those local marshes; shades of lavender indicate seedlings from commercial nurseries. Circles indicate samples from Rumney Marsh Reservation (Rumney), squares indicate samples from the Great Marsh in Rowley (Rowley), diamonds indicate samples from Belle Isle Marsh Reservation (Belle Isle), and triangles indicate samples from commercial nurseries from New Jersey (NJ), Massachusetts (MA), and Maryland (MD).

**FIGURE 5:**
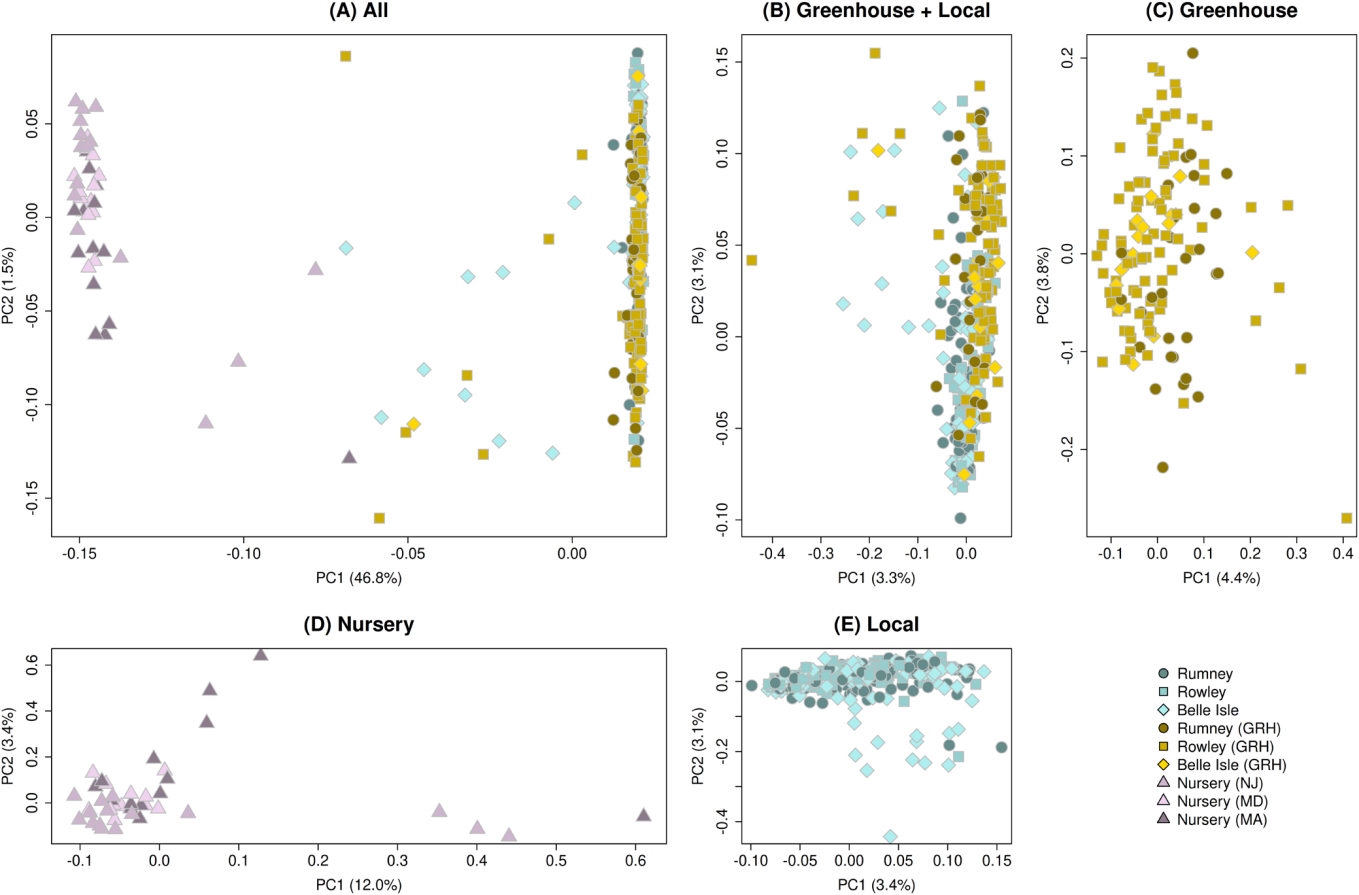
Principal component analysis for *S. patens* for (A) all samples, (B) local marsh samples and the corresponding greenhouse seedlings, (C) greenhouse seedlings only, (D) nursery samples only, and (E) local marshes only. Shades of blue indicate samples from local marshes; shades of gold indicate seedlings produced in the greenhouse from seed from those local source populations; shades of lavender indicate seedlings from commercial nurseries. Circles indicate samples from Rumney Marsh Reservation (Rumney), squares indicate samples from the Great Marsh in Rowley (Rowley), diamonds indicate samples from Belle Isle Marsh Reservation (Belle Isle), and triangles indicate samples from commercial nurseries from New Jersey (NJ), Massachusetts (MA), and Maryland (MD).

Among local populations of *S. alterniflora* , the principal component analysis (PCA) indicated that Belle Isle was more compositionally distinct from Rowley and Rumney, which clustered together (Figure 4E). In contrast, for *S. patens*, Rowley was more differentiated from Rumney and Belle Isle (Figure 5E). Greenhouse samples were differentiated by population in *S. alterniflora* (Figure 4C), but not *S. patens* (Figure 5C). In both species, greenhouse samples generally tended to cluster with their parent local population (Figures 4B and 5B). Nursery samples were compositionally distinct from local and greenhouse samples for both species (Figures 4A and 5A). In addition, the nursery samples for *S. alterniflora* were distinct from one another (Figure 4D), whereas the degree of genetic dissimilarity was lower among nurseries for *S. patens* (Figure 5D).

Analyses of admixture generally confirmed these patterns across a range of K values (Figures S6 and S7), although *S. alterniflora* from Belle Isle and Rumney had more similar patterns of ancestry and were distinct from Rowley, in contrast with the PCA results (Figure S6). The admixture analyses also suggested greater differentiation among *S. patens* greenhouse samples and their paired local population for Rowley and Rumney (Figure S7).

We found a mean pairwise F_ST_ of 0.047 (SD = 0.029) for *S. alterniflora* (Figure S8) and 0.176 (SD = 0.160) for *S. patens* (Figure S9). Consistent with the PCA, F_ST_ values for *S. alterniflora* and *S. patens* indicated that nursery samples were more differentiated from local and greenhouse samples, particularly for *S. patens* (Figures S8 and S9).

The AMOVA indicated a weak and significant difference among the three local populations for *S. alterniflora* (PhiST = 0.008 and *p* = 0.010), and a weak, non‐significant difference in *S. patens* (PhiST = 0.007 and *p* = 0.066). We detected significant differentiation among each pair of local versus greenhouse samples for *S. alterniflora* (Belle PhiST = 0.102; Rowley PhiST = 0.059; Rumney PhiST = 0.048; all comparisons were *p* < 0.001). For *S. patens* , local and greenhouse samples differed for Rowley (PhiST = 0.048, *p* < 0.001) and Rumney (PhiST = 0.121, *p* < 0.001), but not for Belle Isle (PhiST = 0.040, *p* = 0.073). Consistent with all other analyses, there was significant differentiation in both species between nursery and local samples ( *S. alterniflora* PhiST = 0.321, *p* < 0.001; *S. patens* PhiST = 0.121, *p* < 0.001) and between nursery and greenhouse samples ( *S. alterniflora* PhiST = 0.113, *p* < 0.001; *S. patens* PhiST = 0.434, *p* < 0.001).

## 4 Discussion

When restoration involves actively introducing organisms to the restored environment, the source of those organisms becomes a key decision (LaRue et al. 2017; Hendry et al. 2024). Because local adaptation can be common (Hereford 2009), particularly in plants (Leimu and Fischer 2008), the default practice has historically been to use a nearby wild source (local provenancing; Brousseau et al. 2021). An understanding of the genetic diversity and structure of potential sources, and their relationship to the target location, is needed to inform these decisions, particularly given evidence that source selection and resulting eco‐evolutionary dynamics can affect restoration outcomes (Hendry et al. 2024; Truskey et al. 2025). In this study, populations of *S. alterniflora* had reduced relatedness and slightly higher heterozygosity relative to *S. patens* . *S. patens* showed higher differentiation of nursery populations relative to the local and greenhouse populations than *S. alterniflora* , suggesting there are stronger genetic implications of sourcing decisions for *S. patens* in this region. Interestingly, the Rowley and Rumney *S. alterniflora* populations were more similar compositionally, despite Rumney and Belle Isle being much closer geographically. In the context of management and restoration of Belle Isle Marsh Reservation, these results suggest that collections of plants or seed from Rumney could be less preferred, from a genetic perspective, than Belle Isle *S. patens* and *S. alterniflora* populations, because they may disrupt current patterns of genetic structure. Alternatively, supplementation using plants or seed from Rumney or Rowley could mimic gene flow and introduce novel genetic variation to Belle Isle to facilitate its response to ongoing environmental change (Espeland et al. 2017).

Shortages of appropriate source organisms can hinder active restoration efforts (Espeland et al. 2017; Jones et al. 2018), and commercial nurseries play a key role in filling those gaps (White et al. 2018; Allen et al. 2024). The geographic distribution of commercial vendors varies by state, with many vendors clustered near major cities, which can result in geographic gaps between nursery and restoration locations (White et al. 2018; Allen et al. 2024). Although relatively little is known about nursery collection practices, the data that do exist show that those that are collected from the wild (as opposed to sourced from selected lines or other commercial facilities) come from within close proximity to the nursery itself (Engert et al. 2026). Combined with the geographic gaps noted above, these results suggest that nursery sources often may not be very “local” to the restoration site. Consistent with that expectation, when we compared plants from commercial nurseries to those from local and greenhouse populations, nursery plants generally had higher relatedness, higher heterozygosity, and distinct genetic composition relative to local and greenhouse populations for both species. These results are congruent with recent findings that *S. alterniflora* in actively restored marshes in Connecticut that were planted with nursery sources were more similar genetically (based on DNA microsatellites) to those nursery sources and to each other than they were to nearby wild populations (Crosby et al. 2024). Marshes restored with nursery sources also had higher genotypic and allelic diversity than both natural marshes and passively restored marshes that relied on natural recruitment of plants (Sperry et al. 2025), consistent with our finding of higher heterozygosity in nursery plants.

The pattern of equivalent or higher *S. alterniflora* diversity in restored relative to natural marshes has also been found in the Gulf of Mexico (Travis et al. 2002), but runs counter to the reduced genetic diversity found in many restored plant populations (Espeland et al. 2017; Holl et al. 2022). In actively restored marshes, the greater diversity is likely due to the use of seed‐based propagation practices among commercial nurseries in the northeast U.S. as indicated by our interviews with growers (Kollars and Hughes, unpublished data), whereas a mix of sexual and clonal reproduction occurs in wild populations (Travis et al. 2002; Richards et al. 2004; Hughes and Lotterhos 2014). The higher relatedness measured within each of the nursery populations relative to local source and greenhouse populations may also reflect nursery collection and propagation practices (Espeland et al. 2017). For example, if some plants are held over from year to year and propagated clonally (or asexually), then higher relatedness could develop in that stock through time. Alternatively, the nurseries may have collected the seed for their stock from a smaller number of individuals and/or a smaller geographic area than we used, which would likely also contribute to higher relatedness (Espeland et al. 2017; Engert et al. 2026). For example, the substantial overlap in genetic composition of *S. patens* nursery populations suggests that these nurseries may be using a common source, or that their sources are not genetically differentiated. Greater communication and collaboration among scientists, practitioners, and commercial growers can help to clarify these uncertainties and build a more robust plant supply (White et al. 2018; Hughes et al. 2019; Truskey et al. 2025).

As mentioned above, commercial stocks often have lower genetic diversity and/or effective population size than wild populations, particularly for species with high fecundity and high variance in reproductive success (Hedgecock and Pudovkin 2011; Zhang et al. 2017; Hughes et al. 2019), even if they are not being actively selected for a particular trait (Morvezen et al. 2016). Although it is generally unclear when in the cultivation process this decline occurs, hatchery production of shellfish indicates it can happen in as little as a single generation (Hughes et al. 2019; Sotka et al. 2025). We found some evidence for change in genetic diversity and composition during our propagation process, with greenhouse populations having consistently lower heterozygosity than their local counterparts. However, relatedness was generally equivalent or lower in greenhouse plants relative to their source, suggesting that our greenhouse populations did not skew towards a few highly related families that were particularly successful in the greenhouse environment (as in sweepstakes reproduction; Hedgecock and Pudovkin 2011). Further, although the AMOVA indicated genetic differentiation between paired local and greenhouse populations, the PCA and ancestry analyses indicated that genetic composition was similar, suggesting that greenhouse populations were broadly representative of their respective local populations. Thus, publicly available methods for local seed collection, germination, and propagation practices for both *S. alterniflora* and *S. patens* appear to offer a means of generating plant stocks for restoration that can reflect genetic diversity and composition of local, wild marshes.

To our knowledge, this is the first assessment of *S. patens* genetic variation using a SNP‐sequencing approach, and among the first for *S. alterniflora* in this region. Not surprisingly given that the two species are not close congeners (~10% substitutions per site at an internal transcribed spacer region; Peterson et al. 2014), more reads of *S. alterniflora* than *S. patens* mapped to the *S. alterniflora* genome, and there was little overlap in the SNP sets for the two species. Despite the greater number of hard‐called SNPs and genotype likelihoods for *S. alterniflora* , we found that *S. patens* had significantly higher levels of heterozygosity, suggesting greater genetic diversity. In contrast, relatedness for *S. patens* was substantially higher on average than *S. alterniflora* , with mean pairwise relatedness estimates in source and nursery populations consistent with half siblings. These genetic differences between *S. alterniflora* and *S. patens* were somewhat surprising: although they are not close congeners, the two species do share many life history traits. For example, both species are rhizomatous, perennial grasses. In addition, both species are capable of clonal and sexual reproduction, with sexual reproduction occurring via wind pollination in the summer and early fall months (Silander and Antonovics 1979; Daehler and Strong 1994). Further, they share similar ranges across the Atlantic Coast of the U.S. and Canada, and throughout much of the Gulf of Mexico. Although both species are capable of selfing (Silander and Antonovics 1979; Daehler and Strong 1994), the reduced relatedness of *S. alternilfora* relative to *S. patens* suggests that inbreeding may be more common in *S. patens* , at least in our study region. Relatively little is known about dispersal distances of either species, but the increased genetic differentiation (measured as pairwise F_ST_) in *S. alterniflora* compared to *S. patens* suggests that realized dispersal may vary. Regardless of the underlying mechanisms, the observed differences suggest caution is needed regarding making inferences from one species to the other.

Evolutionary theory and a substantial body of small‐scale experimental studies, including with marsh plants (Hughes 2014; Zerebecki et al. 2017, 2021; Noto and Hughes 2020; Zogg and Travis 2022; Zerebecki and Hughes 2023), suggest that genetic diversity and composition can influence the ecosystem functions and services that plant populations provide to people, as well as their long‐term persistence and resilience (Hughes et al. 2008; Espeland et al. 2017; Bolnick et al. 2011; Schäfer et al. 2020). Thus, the genetic differences between nursery and local/greenhouse populations documented here, which are consistent with recent studies of the genetics of restored marshes (Crosby et al. 2024; Sperry et al. 2025), demonstrate that sourcing decisions have the potential to impact the success of marsh restoration efforts, as in other systems (Hendry et al. 2024; Truskey et al. 2025). Achieving successful ecological restoration outcomes rely on the survival of salt marsh transplants. A recent global meta‐analysis by Lui et al. (2024) found that salt marsh plantings have an average survival rate of 53% (*n* = 210 studies). Therefore, better understanding the differences between commercial and local plant sources could enhance post‐transplant survival and maximize restoration benefits. Additional work is needed to document the ecological outcomes associated with these genetic differences, particularly in light of increasing calls for restoration strategies (e.g., assisted gene flow) that involve sourcing from greater distances and distinct regions.

Despite the evidence that plant genetic variation impacts populations and communities, coastal restoration practitioners view genetic diversity as a less important restoration strategy for salt marshes than for other coastal systems (e.g., vs. coral reefs or oyster reefs; Hughes et al. 2020). Several knowledge gaps may limit the perceived relevance of genetic variation as a key component of marsh restoration practice, including a general lack of clarity of the differences in diversity and composition across sourcing strategies such as those demonstrated here. Even with a clear understanding of system dynamics, practitioner decisions regarding sourcing strategies are likely influenced and constrained by numerous factors, including funding, policies (e.g., permits, regulations), availability of plant source material, and project timelines. Policies that incentivize or require sourcing decisions that preserve genetic and trait variation could accelerate both research and adoption of these practices (Kochnower et al. 2015; Pierson et al. 2016). Further, successes in systems such as the tallgrass prairie (White et al. 2018) highlight the value of collaboration among practitioners, scientists, commercial growers, and policy makers for generating a robust and genetically diverse plant supply and increasing the transparency of sourcing decisions for future restoration efforts.

## Funding

This work was supported by funding from the U.S. Army Corps of Engineers (USACE) Engineer Research and Development Center (ERDC), Engineering With Nature program and Ramboll Americas Engineering Solutions.

## Ethics Statement

The manuscript has not been submitted elsewhere. All research meets the ethical guidelines of the U.S.

## Conflicts of Interest

The authors declare no conflicts of interest.

## Supporting information


**Figure S1:** Our dataset for *Spartina alterniflora* hard‐called genotypes included 4213 SNPs across 322 individuals. All have minor allele frequencies (MAF) > 5%. Top panel: MAF histogram. Bottom panel: Observed vs. Expected heterozygosity (with 1:1 line).
**Figure S2:** Our dataset for *Spartina patens* hard‐called genotypes included 582 SNPs across 350 individuals. All have minor allele frequencies (MAF) > 5%. Top panel: MAF histogram. Bottom panel: Observed vs. Expected heterozygosity (with 1:1 line).
**Figure S3:** Average reads per individual × SNP combination. Top panel: *S. alterniflora* : 7008 SNPs; mean = 3.03; range = [1.5, 33.0]. Bottom panel: *S. patens* : 5695 SNPs; mean = 4.38; range = [1.0, 133.5].
**Figure S4:** Pairwise relatedness between individuals from local, nursery, and greenhouse sources for (A) *S. alterniflora* and (B) *S. patens.*

**Figure S5:** Heterozygosity between individuals from local, nursery, and greenhouse sources for (A) *S. alterniflora* (clone‐corrected) and (B) *S. patens.*

**Figure S6:** Admixture plot from ngsadmix for cluster K3, K4, and K5 for *S. alterniflora* .
**Figgure S7**. Admixture plot from ngsadmix for cluster K3, K4, and K5 for *S. patens* .
**Figure S8:** Pairwise F_ST_ for *S. alterniflora* for nursery, local, and greenhouse samples.
**Figure S9:** Pairwise F_ST_ for *S. patens* for nursery, local, and greenhouse samples.
**Table S1:** Table of putative clonal pairs. Samples in bold and italics were removed from analyses that excluded clones.
**Table S2:** Source summary for relatedness for *Spartina alterniflora* .
**Table S3:** Site summary for relatedness for *Spartina alterniflora* .
**Table S4:** Source summary for relatedness for *Spartina patens* .
**Table S5:** Site summary for relatedness for *Spartina patens* .
**Table S6:** Linear model summary for clone‐corrected *S. alterniflora* heterozygosity estimates among sites.
**Table S7:** Linear model summary for *S. patens* heterozygosity estimates among sites.

## Data Availability

Data for this study are available at Github: https://github.com/northeastern‐rc‐internal/seedpod.
