# Supplementary material for: Genetic Implications of Plant Sourcing Strategies for Salt Marsh Restoration
Source: Evol Appl. 2026 Jul 29;19(8):e70292. doi: 10.1111/eva.70292 (PMC13417026; doi:10.1111/eva.70292)

**Title: Genetic implications of plant sourcing strategies for salt marsh restoration**

**Table S1**. Table of putative clonal pairs. Samples in bold and italics were removed from analyses that excluded clones.

| **Sample 1** | | | | **Sample 2** | | | |
| --- | --- | --- | --- | --- | --- | --- | --- |
| **Site type** | **Site name** | **Subsite** | **Rep** | **Site type** | **Site name** | **Subsite** | **Rep** |
| ***Local*** | ***Rowley*** | ***Law’s*** | ***A06*** | Local | Rowley | Law’s | B05 |
| Local | Belle Isle | Key | B09 | ***Local*** | ***Belle Isle*** | ***Key*** | ***C09*** |
| ***Local*** | ***Belle Isle*** | ***Key*** | ***C09*** | ***Local*** | ***Belle Isle*** | ***Key*** | ***H08*** |
| ***Local*** | ***Belle Isle*** | ***Key*** | ***C09*** | Local | Belle Isle | L-Berm | H04 |
| ***Local*** | ***Rowley*** | ***Laws*** | ***D04*** | Local | Rowley | Law’s | G03 |
| ***Local*** | ***Belle Isle*** | ***Rosie’s Pond*** | ***E06*** | Local | Belle Isle | Rosie’s Pond | H12 |
| ***Local*** | ***Belle Isle*** | ***Key*** | ***H08*** | Local | Belle Isle | Rosie’s Pond | A03 |
| ***Local*** | ***Belle Isle*** | ***Key*** | ***H08*** | Local | Belle Isle | L-Berm | H04 |
| ***Local*** | ***Belle Isle*** | ***Rosie’s Pond*** | ***C03*** | Local | Belle Isle | L-Berm | G09 |
| ***Local*** | ***Rumney*** | ***Far*** | ***D08*** | Local | Rumney | Far | E08 |
| ***Local*** | ***Rumney*** | ***Far*** | ***D08*** | Greenhouse | Rowley | Stackyard | H12 |

**Table S2.** Source summary for relatedness for *Spartina alterniflora.*

Df Sum Sq Mean Sq F value Pr(>F)

Source 2 4.14 2.0722 70.84 <2e-16 ***

Residuals 8009 234.26 0.0292

---

Signif. codes: 0 ‘***’ 0.001 ‘**’ 0.01 ‘*’ 0.05 ‘.’ 0.1 ‘ ’ 1

Tukey multiple comparisons of means

95% family-wise confidence level

diff lwr upr p adj

Local-Nursery -0.15686355 -0.189129181 -0.12459791 **0.0e+00**

Greenhouse-Nursery -0.13809837 -0.170519426 -0.10567732 **0.0e+00**

Greenhouse-Local 0.01876517 0.009649519 0.02788083 **4.2e-06**

**Table S3.** Site summary for relatedness for *Spartina alterniflora*.

Df Sum Sq Mean Sq F value Pr(>F)

Source sites 8 6.64 0.8296 28.64 **<2e-16 *****

Residuals 8003 231.77 0.0290

---

Signif. codes: 0 ‘***’ 0.001 ‘**’ 0.01 ‘*’ 0.05 ‘.’ 0.1 ‘ ’ 1

Tukey multiple comparisons of means

95% family-wise confidence level

diff lwr upr p adj

Nursery (MA)-Nursery (MD) -0.122867767 -0.241947992 -0.0037875423 **0.0370476**

Nursery (NJ)-Nursery (MD) -0.084367439 -0.176278914 0.0075440351 0.1019337

Belle Isle-Nursery (MD) -0.198767935 -0.265241017 -0.1322948522 **0.0000000**

Belle Isle(GRH)-Nursery(MD)-0.114071078 -0.194984503 -0.0331576531 **0.0004208**

Rowley-Nursery (MD) -0.221164517 -0.287584293 -0.1547447410 **0.0000000**

Rowley (GRH)-Nursery (MD) -0.183785133 -0.249865398 -0.1177048685 **0.0000000**

Rumney-Nursery (MD) -0.219044867 -0.285464643 -0.1526250912 **0.0000000**

Rumney (GRH)-Nursery(MD) -0.216443918 -0.282973336 -0.1499144998 **0.0000000**

Nursery (NJ)-Nursery (MA) 0.038500328 -0.080579897 0.1575805529 0.9857061

Belle Isle-Nursery (MA) -0.075900167 -0.176652594 0.0248522596 0.3196731

Belle Isle(GRH)-Nursery(MA) 0.008796689 -0.102015599 0.1196089773 0.9999996

Rowley-Nursery (MA) -0.098296750 -0.199014015 0.0024205153 0.0621700

Rowley (GRH)-Nursery (MA) -0.060917366 -0.161411058 0.0395763267 0.6272018

Rumney-Nursery (MA) -0.096177100 -0.196894365 0.0045401652 0.0747675

Rumney(GRH)-Nursery(MA) -0.093576150 -0.194365754 0.0072134537 0.0935207

Belle Isle-Nursery (NJ) -0.114400495 -0.180873578 -0.0479274128 **0.0000034**

Belle Isle(GRH)-Nursery(NJ) -0.029703639 -0.110617064 0.0512097863 0.9683022

Rowley-Nursery (NJ) -0.136797078 -0.203216854 -0.0703773016 **0.0000000**

Rowley (GRH)-Nursery (NJ) -0.099417694 -0.165497958 -0.0333374291 **0.0001076**

Rumney-Nursery (NJ) -0.134677428 -0.201097204 -0.0682576518 **0.0000000**

Rumney (GRH)-Nursery (NJ) -0.132076478 -0.198605896 -0.0655470605 **0.0000000**

Belle Isle (GRH)-Belle Isle 0.084696857 0.034517838 0.1348758752 **0.0000060**

Rowley-Belle Isle -0.022396582 -0.041955167 -0.0028379980 **0.0114948**

Rowley (GRH)-Belle Isle 0.014982802 -0.003389812 0.0333554153 0.2175206

Rumney-Belle Isle -0.020276933 -0.039835517 -0.0007183481 0.0353275

Rumney (GRH)-Belle Isle -0.017675983 -0.037603727 0.0022517616 0.1301711

Rowley-Belle Isle (GRH) -0.107093439 -0.157201820 -0.0569850581 **0.0000000**

Rowley(GRH)-Belle Isle(GRH)-0.069714055 -0.119371527 -0.0200565826 **0.0004567**

Rumney-Belle Isle (GRH) -0.104973789 -0.155082170 -0.0548654082 **0.0000000**

Rumney(GRH)-Belle Isle(GRH)-0.102372840-0.152626463-0.0521192166 **0.0000000**

Rowley (GRH)-Rowley 0.037379384 0.019200581 0.0555581875 **0.0000000**

Rumney-Rowley 0.002119650 -0.017256990 0.0214962900 0.9999951

Rumney (GRH)-Rowley 0.004720599 -0.015028602 0.0244698010 0.9981836

Rumney-Rowley (GRH) -0.035259734 -0.053438538 -0.0170809310 **0.0000001**

Rumney(GRH)-Rowley(GRH)-0.032658785 -0.051234190 -0.0140833798 **0.0000018**

Rumney (GRH)-Rumney 0.002600950 -0.017148252 0.0223501512 0.9999791

**Table S4.** Source summary for relatedness for *Spartina patens.*

Df Sum Sq Mean Sq F value Pr(>F)

Source 2 76.03 38.01 1526 <2e-16 ***

Residuals 9675 240.96 0.02

---

Signif. codes: 0 ‘***’ 0.001 ‘**’ 0.01 ‘*’ 0.05 ‘.’ 0.1 ‘ ’ 1

Tukey multiple comparisons of means

95% family-wise confidence level

diff lwr upr p adj

Local-Nursery -0.3187782 -0.3417350 -0.2958213 **0**

Greenhouse-Nursery -0.4458175 -0.4688446 -0.4227905 **0**

Greenhouse-Local -0.1270393 -0.1346804 -0.1193983 **0**

**Table S5.** Site summary for relatedness for *Spartina patens.*

Df Sum Sq Mean Sq F value Pr(>F)

Source site 8 99.48 12.435 552.8 **<2e-16 *****

Residuals 9669 217.51 0.022

---

Signif. codes: 0 ‘***’ 0.001 ‘**’ 0.01 ‘*’ 0.05 ‘.’ 0.1 ‘ ’ 1

Tukey multiple comparisons of means

95% family-wise confidence level

diff lwr upr p adj

Nursery (MA)-Nursery (MD) -0.017888799 -0.089689064 0.05391147 0.9975631

Nursery (NJ)-Nursery (MD) 0.003966694 -0.065589257 0.07352264 1.0000000

Belle Isle-Nursery (MD) -0.416700929 -0.470660027 -0.36274183 **0.0000000**

Belle Isle(GRH)-Nursery(MD) -0.405216796 -0.472894461 -0.33753913 **0.0000000**

Rowley-Nursery (MD) -0.285239021 -0.339198119 -0.23127992 **0.0000000**

Rowley (GRH)-Nursery (MD) -0.459206713 -0.512415834 -0.40599759 **0.0000000**

Rumney-Nursery (MD) -0.273117815 -0.326953190 -0.21928244 **0.0000000**

Rumney(GRH)-Nursery(MD) -0.377045224 -0.434571077 -0.31951937 **0.0000000**

Nursery (NJ)-Nursery (MA) 0.021855492 -0.044788866 0.08849985 0.9843419

Belle Isle-Nursery (MA) -0.398812130 -0.448962149 -0.34866211 **0.0000000**

Belle Isle(GRH)-Nursery(MA) -0.387327997 -0.452009575 -0.32264642 **0.0000000**

Rowley-Nursery (MA) -0.267350222 -0.317500241 -0.21720020 **0.0000000**

Rowley (GRH)-Nursery (MA) -0.441317915 -0.490660095 -0.39197573 **0.0000000**

Rumney-Nursery (MA) -0.255229016 -0.305245891 -0.20521214 **0.0000000**

Rumney(GRH)-Nursery(MA) -0.359156426 -0.413125526 -0.30518733 **0.0000000**

Belle Isle-Nursery (NJ) -0.420667623 -0.467548072 -0.37378717 **0.0000000**

Belle Isle(GRH)-Nursery(NJ) -0.409183489 -0.471364344 -0.34700264 **0.0000000**

Rowley-Nursery (NJ) -0.289205715 -0.336086164 -0.24232527 **0.0000000**

Rowley (GRH)-Nursery (NJ) -0.463173407 -0.509188653 -0.41715816 **0.0000000**

Rumney-Nursery (NJ) -0.277084509 -0.323822501 -0.23034652 **0.0000000**

Rumney (GRH)-Nursery (NJ) -0.381011918 -0.431957143 -0.33006669 **0.0000000**

Belle Isle (GRH)-Belle Isle 0.011484133 -0.032561441 0.05552971 0.9966470

Rowley-Belle Isle 0.131461908 0.114989797 0.14793402 **0.0000000**

Rowley (GRH)-Belle Isle -0.042505784 -0.056324495 -0.02868707 **0.0000000**

Rumney-Belle Isle 0.143583114 0.127520927 0.15964530 **0.0000000**

Rumney (GRH)-Belle Isle 0.039655704 0.013791253 0.06552016 **0.0000698**

Rowley-Belle Isle (GRH) 0.119977774 0.075932201 0.16402335 **0.0000000**

Rowley(GRH)-Belle Isle(GRH)-0.053989918-0.097113449 -0.01086639 **0.0033154**

Rumney-Belle Isle (GRH) 0.132098981 0.088205063 0.17599290 **0.0000000**

Rumney(GRH)-Belle Isle(GRH)0.028171571-0.020177700 0.07652084 0.6771708

Rowley (GRH)-Rowley -0.173967692 -0.187786403 -0.16014898 **0.0000000**

Rumney-Rowley 0.012121206 -0.003940981 0.02818339 0.3173242

Rumney (GRH)-Rowley -0.091806203 -0.117670655 -0.06594175 **0.0000000**

Rumney-Rowley (GRH) 0.186088898 0.172761476 0.19941632 **0.0000000**

Rumney(GRH)-Rowley(GRH)0.082161489 0.057900488 0.10642249 **0.0000000**

Rumney (GRH)-Rumney -0.103927410 -0.129532746 -0.07832207 **0.0000000**

**Table S6.** Linear model summary for clone-corrected *S. alterniflora* heterozygosity estimates among sites.

Df Sum Sq Mean Sq F value Pr(>F)

Source site 8 0.2862 0.03578 6.736 3.67e-08 ***

Residuals 318 1.6892 0.00531

---

Signif. codes: 0 ‘***’ 0.001 ‘**’ 0.01 ‘*’ 0.05 ‘.’ 0.1 ‘ ’ 1

Tukey multiple comparisons of means

95% family-wise confidence level

diff lwr upr p adj

Belle Isle (GRH)-Belle Isle -0.003895782 -0.0686891385 0.060897575 1.0000000

Nur_ND-Belle Isle -0.023795992 -0.0990971733 0.051505189 0.9868735

Nur_MA-Belle Isle -0.063328578 -0.1495653127 0.022908156 0.3491040

Nur_NJ-Belle Isle 0.005060359 -0.0675874958 0.077708214 0.9999998

Rowley-Belle Isle -0.019081445 -0.0626901669 0.024527276 0.9094721

Rowley (GRH)-Belle Isle -0.051273500 -0.0934882388 -0.009058761 **0.0055051**

Rumney-Belle Isle 0.023886506 -0.0197222160 0.067495227 0.7396104

Rumney (GRH)-Belle Isle -0.056419066 -0.1004335288 -0.012404603 **0.0024949**

Nur_MD-Belle Isle (GRH) -0.019900211 -0.1090589419 0.069258521 0.9988032

Nur_MA-Belle Isle (GRH) -0.059432796 -0.1580014826 0.039135890 0.6258526

Nur_NJ-Belle Isle (GRH) 0.008956141 -0.0779732692 0.095885551 0.9999966

Rowley-Belle Isle (GRH) -0.015185664 -0.0798442446 0.049472918 0.9982753

Rowley (GRH)-Belle Isle (GRH) -0.047377718 -0.1111044448 0.016349008 0.3320411

Rumney-Belle Isle (GRH) 0.027782287 -0.0368762937 0.092440868 0.9178243

Rumney (GRH)-Belle Isle (GRH) -0.052523284 -0.1174562069 0.012409638 0.2233449

Nur_MA-Nur_MD -0.039532586 -0.1453053358 0.066240164 0.9626924

Nur_NJ-Nur_MD 0.028856352 -0.0661637047 0.123876408 0.9898800

Rowley-Nur_MD 0.004714547 -0.0704706966 0.079899791 0.9999999

Rowley (GRH)-Nur_MD -0.027477508 -0.1018628855 0.046907870 0.9652110

Rumney-Nur_MD 0.047682498 -0.0275027457 0.122867742 0.5579455

Rumney (GRH)-Nur_MD -0.032623074 -0.1080443783 0.042798231 0.9148468

Nur_NJ-Nur_MA 0.068388937 -0.0355115806 0.172289455 0.5056352

Rowley-Nur_MA 0.044247133 -0.0418883847 0.130382651 0.8019591

Rowley (GRH)-Nur_MA 0.012055078 -0.0733831508 0.097493307 0.9999613

Rumney-Nur_MA 0.087215084 0.0010795662 0.173350601 **0.0445916**

Rumney (GRH)-Nur_MA 0.006909512 -0.0794321332 0.093251158 0.9999995

Rowley-Nur_NJ -0.024141805 -0.0966694811 0.048385872 0.9816869

Rowley (GRH)-Nur_NJ -0.056333859 -0.1280320283 0.015364310 0.2588681

Rumney-Nur_NJ 0.018826146 -0.0537015302 0.091353823 0.9965092

Rumney (GRH)-Nur_NJ -0.061479425 -0.1342517841 0.011292934 0.1749942

Rowley (GRH)-Rowley -0.032192055 -0.0741996398 0.009815530 0.2911069

Rumney-Rowley 0.042967951 -0.0004402698 0.086376172 0.0547769

Rumney (GRH)-Rowley -0.037337621 -0.0811534393 0.006478198 0.1661284

Rumney-Rowley (GRH) 0.075160006 0.0331524205 0.117167591 **0.0000018**

Rumney (GRH)-Rowley (GRH) -0.005145566 -0.0475742066 0.037283074 0.9999880

Rumney (GRH)-Rumney -0.080305572 -0.1241213902 -0.036489753 **0.0000009**

**Table S7.** Linear model summary for *S. patens* heterozygosity estimates among sites.

Df Sum Sq Mean Sq F value Pr(>F)

Site 8 0.421 0.0526 8.285 <2.85e-10 ***

Residuals 341 2.166 0.0063

---

Signif. codes: 0 ‘***’ 0.001 ‘**’ 0.01 ‘*’ 0.05 ‘.’ 0.1 ‘ ’ 1

Tukey multiple comparisons of means

95% family-wise confidence level

diff lwr upr p adj

Belle Isle (GRH)-Belle Isle -0.039912390 -0.11030283 0.030478046 0.7018821

Nur_MD-Belle Isle 0.028201325 -0.04826841 0.104671056 0.9656827

Nur_MA-Belle Isle 0.032766574 -0.04144598 0.106979128 0.9053683

Nur_NJ-Belle Isle 0.090803105 0.01860383 0.163002380 **0.0033154**

Rowley-Belle Isle -0.029401676 -0.07600611 0.017202755 0.5657812

Rowley (GRH)-Belle Isle -0.050726310 -0.09293420 -0.008518420 **0.0063422**

Rumney-Belle Isle -0.027601730 -0.07361992 0.018416459 0.6332156

Rumney (GRH)-Belle Isle -0.061206243 -0.11795580 -0.004456691 **0.0236428**

Nur_MD-Belle Isle (GRH) 0.068113715 -0.02478655 0.161013985 0.3517582

Nur_MA-Belle Isle (GRH) 0.072678965 -0.01837236 0.163730288 0.2395651

Nur_NJ-Belle Isle (GRH) 0.130715495 0.04129751 0.220133479 **0.0002400**

Rowley-Belle Isle (GRH) 0.010510714 -0.05987972 0.080901150 0.9999406

Rowley (GRH)-Belle Isle (GRH) -0.010813920 -0.07837387 0.056746027 0.9998991

Rumney-Belle Isle (GRH) 0.012310660 -0.05769301 0.082314333 0.9997947

Rumney (GRH)-Belle Isle (GRH) -0.021293853 -0.09877516 0.056187449 0.9948500

Nur_MA-Nur_MD 0.004565250 -0.09126350 0.100394002 1.0000000

Nur_NJ-Nur_MD 0.062601780 -0.03167644 0.156879996 0.4936618

Rowley-Nur_MD -0.057603001 -0.13407273 0.018866729 0.3144959

Rowley (GRH)-Nur_MD -0.078927635 -0.15280018 -0.005055092 **0.0261394**

Rumney-Nur_MD -0.055803055 -0.13191692 0.020310810 0.3518336

Rumney (GRH)-Nur_MD -0.089407568 -0.17245068 -0.006364457 **0.0240937**

Nur_NJ-Nur_MA 0.058036531 -0.03442030 0.150493360 0.5726876

Rowley-Nur_MA -0.062168251 -0.13638080 0.012044302 0.1841261

Rowley (GRH)-Nur_MA -0.083492885 -0.15502635 -0.011959424 **0.0093313**

Rumney-Nur_MA -0.060368304 -0.13421411 0.013477506 0.2112699

Rumney (GRH)-Nur_MA -0.093972818 -0.17494221 -0.013003430 **0.0100284**

Rowley-Nur_NJ -0.120204782 -0.19240406 -0.048005507 **0.0000123**

Rowley (GRH)-Nur_NJ -0.141529415 -0.21097197 -0.072086862 **0.0000000**

Rumney-Nur_NJ -0.118404835 -0.19022709 -0.046582583 **0.0000158**

Rumney (GRH)-Nur_NJ -0.152009349 -0.23113756 -0.072881136 **0.0000002**

Rowley (GRH)-Rowley -0.021324634 -0.06353252 0.020883256 0.8166666

Rumney-Rowley 0.001799946 -0.04421824 0.047818135 1.0000000

Rumney (GRH)-Rowley -0.031804567 -0.08855412 0.024944986 0.7151499

Rumney-Rowley (GRH) 0.023124580 -0.01843510 0.064684256 0.7231537

Rumney (GRH)-Rowley (GRH) -0.010479933 -0.06367810 0.042718231 0.9995219

Rumney (GRH)-Rumney -0.033604513 -0.08987362 0.022664594 0.6387614

**Figures**

**Figure S1**. Our dataset for *Spartina alterniflora* hard-called genotypes included 4213 SNPs across 322 individuals. All have minor allele frequencies (MAF) >5%. Top panel: MAF histogram. Bottom panel: Observed vs Expected heterozygosity (with 1:1 line).


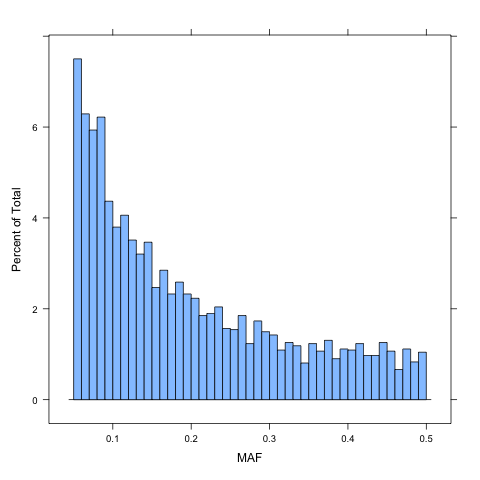


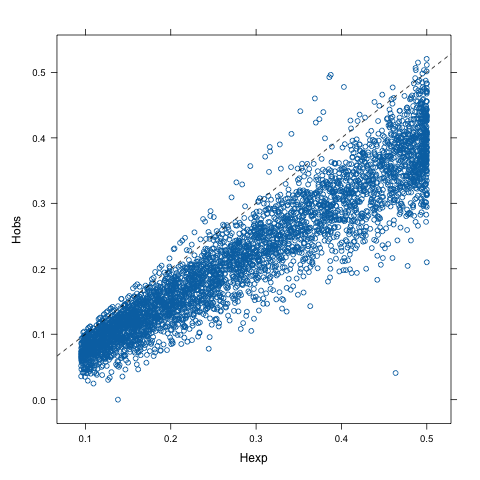


**Figure S2.** Our dataset for *Spartina patens* hard-called genotypes included 582 SNPs across 350 individuals. All have minor allele frequencies (MAF) >5%. Top panel: MAF histogram. Bottom panel: Observed vs Expected heterozygosity (with 1:1 line).


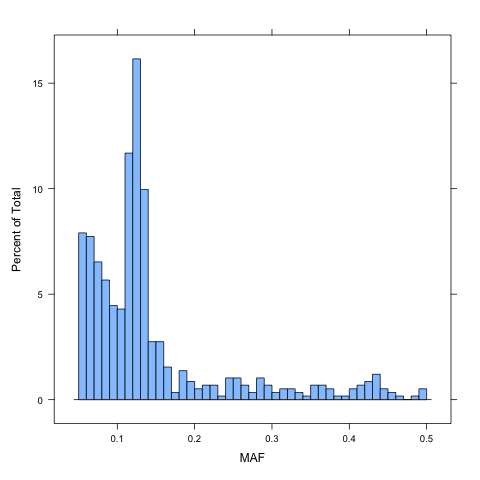


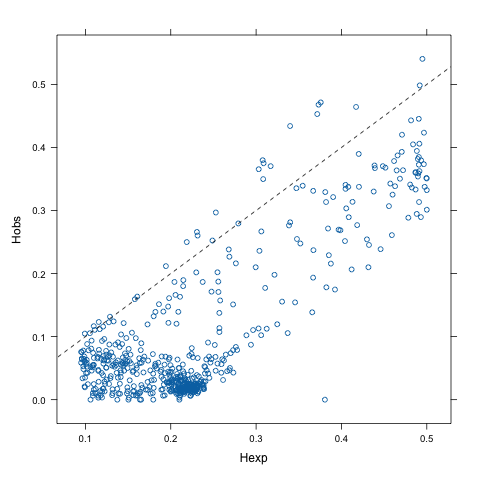


**Figure S3**. Average reads per individual x SNP combination. Top panel: *S. alterniflora*: 7,008 SNPs; mean = 3.03; range = [1.5, 33.0]. Bottom panel: *S. patens*: 5,695 SNPs; mean = 4.38; range = [1.0, 133.5].


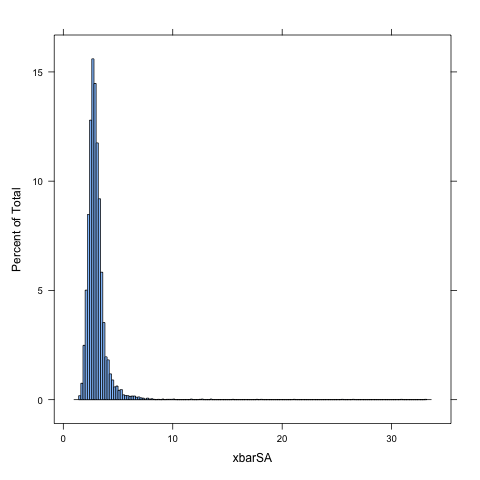


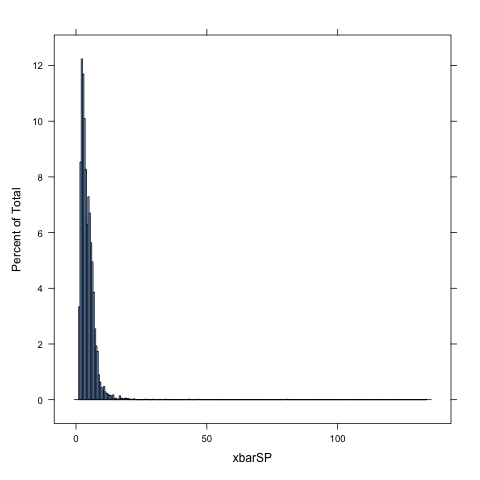


**Figure S4**. Pairwise relatedness between individuals from local, nursery, and greenhouse sources for (A) *S. alterniflora* and (B) *S. patens*


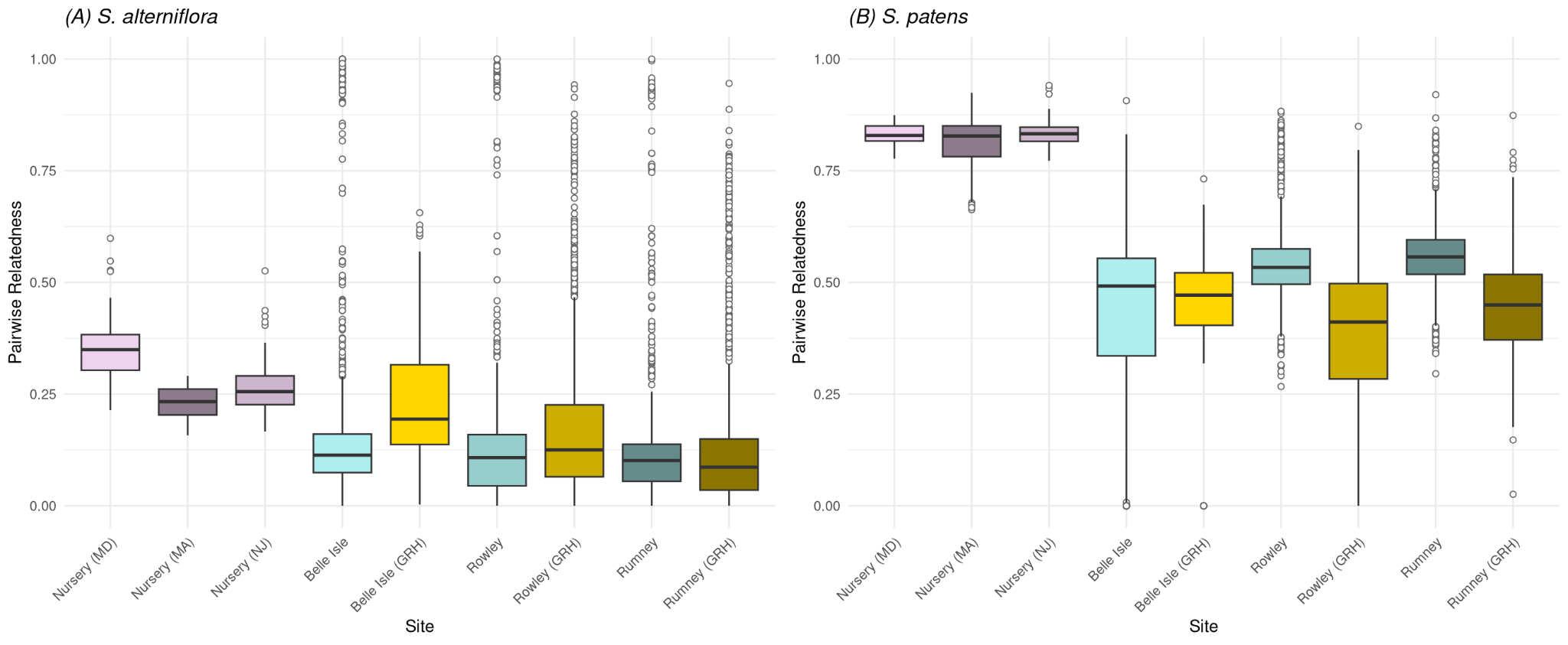


**Figure S5**. Heterozygosity between individuals from local, nursery, and greenhouse sources for (A) *S. alterniflora* (clone-corrected) and (B) *S. patens*

*
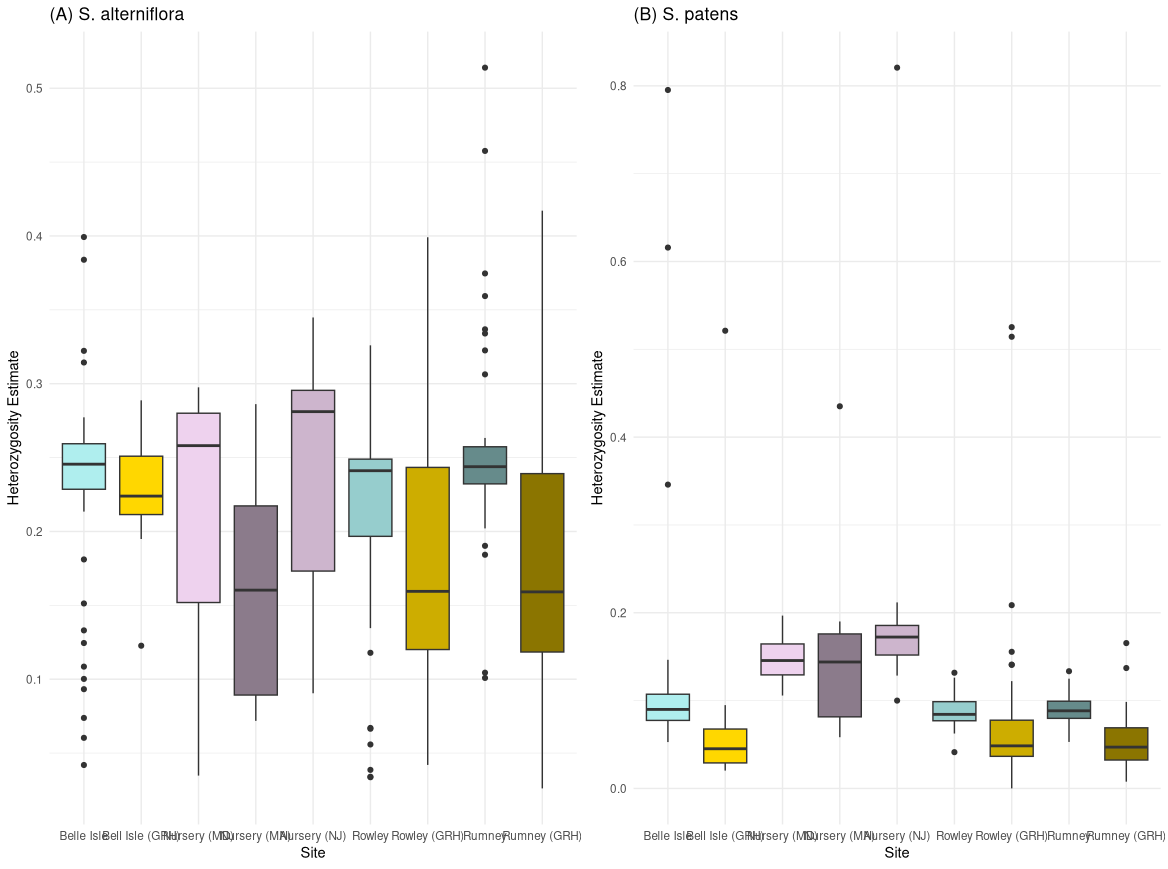
*

**Fig. S6.** Admixture plot from ngsadmix for cluster K3, K4, and K5 for *S. alterniflora.*


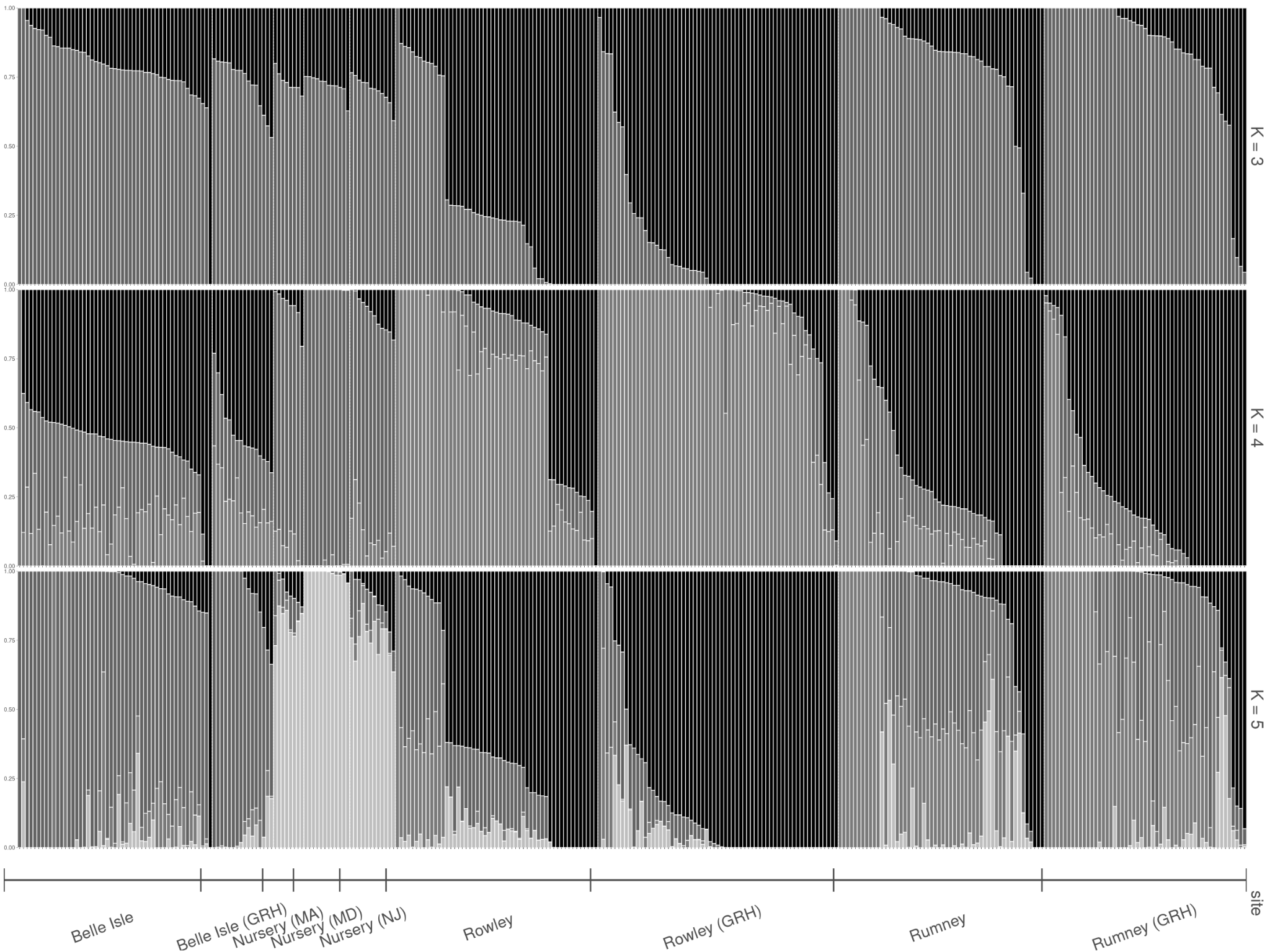


**Fig. S7**. Admixture plot from ngsadmix for cluster K3, K4, and K5 for *S. patens.*

*
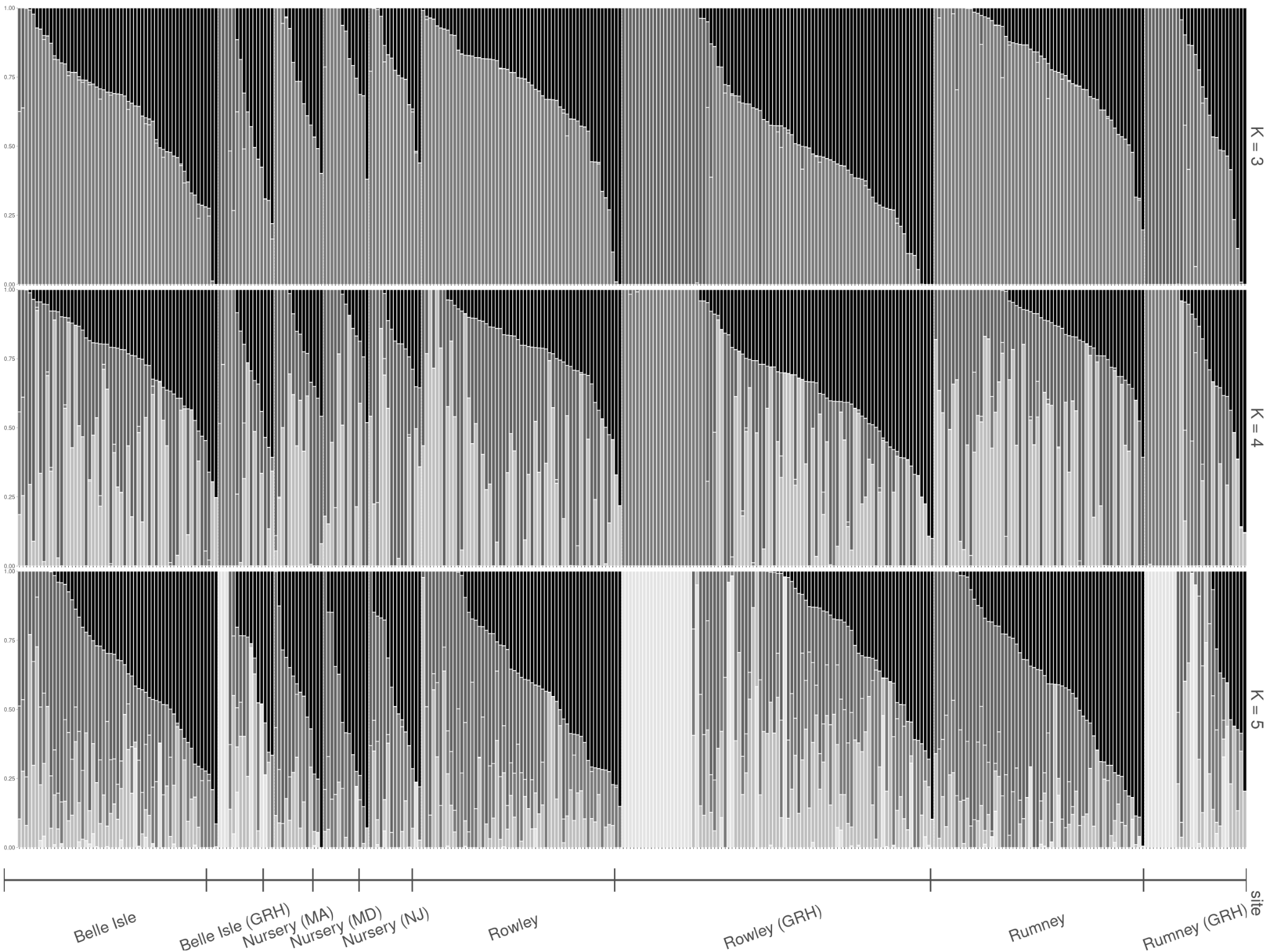
*

**Figure S8**. Pairwise F_ST_ for *S. alterniflora* for nursery, local, and greenhouse samples.


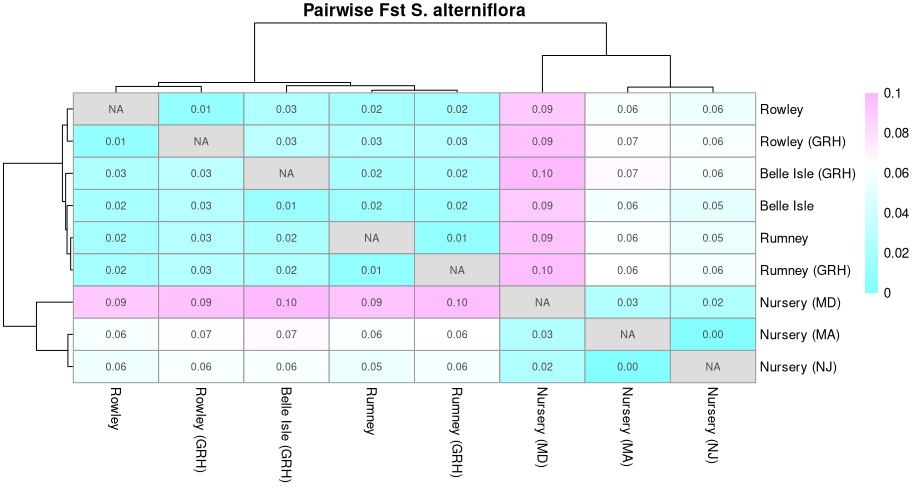


**Figure S9**. Pairwise F_ST_ for *S. patens* for nursery, local, and greenhouse samples.


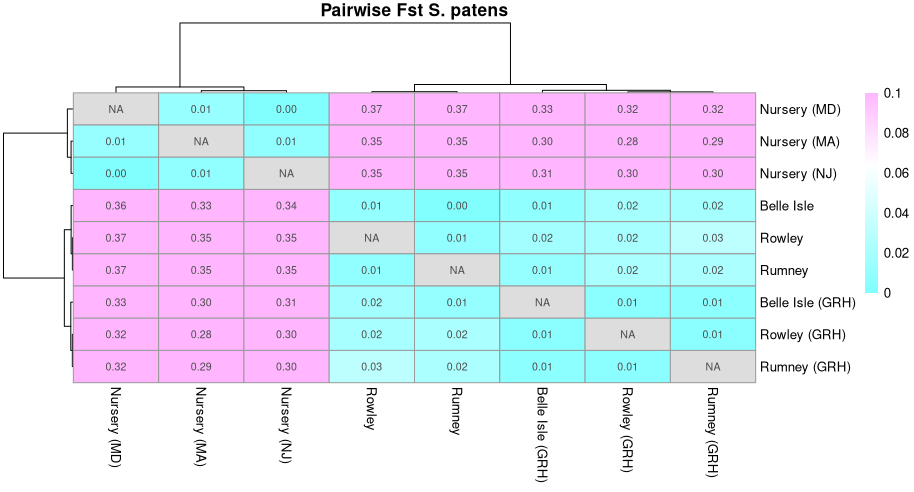

Supplement: Supplementary file 1 — Figure S1: Our dataset for Spartina alterniflora hard‐called genotypes included 4213 SNPs across 322 individuals. All have minor allele frequencies (MAF) > 5%. Top panel: MAF histogram. Bottom panel: Observed vs. Expected heterozygosity (with 1:1 line). Figure S2: Our dataset for Spartina patens hard‐called genotypes included 582 SNPs across 350 individuals. All have minor allele frequencies (MAF) > 5%. Top panel: MAF histogram. Bottom panel: Observed vs. Expected heterozygosity (with 1:1 line). Figure S3: Average reads per individual × SNP combination. Top panel: S. alterniflora : 7008 SNPs; mean = 3.03; range = [1.5, 33.0]. Bottom panel: S. patens : 5695 SNPs; mean = 4.38; range = [1.0, 133.5]. Figure S4: Pairwise relatedness between individuals from local, nursery, and greenhouse sources for (A) S. alterniflora and (B) S. patens. Figure S5: Heterozygosity between individuals from local, nursery, and greenhouse sources for (A) S. alterniflora (clone‐corrected) and (B) S. patens. Figure S6: Admixture plot from ngsadmix for cluster K3, K4, and K5 for S. alterniflora . Figgure S7. Admixture plot from ngsadmix for cluster K3, K4, and K5 for S. patens . Figure S8: Pairwise FST for S. alterniflora for nursery, local, and greenhouse samples. Figure S9: Pairwise FST for S. patens for nursery, local, and greenhouse samples. Table S1: Table of putative clonal pairs. Samples in bold and italics were removed from analyses that excluded clones. Table S2: Source summary for relatedness for Spartina alterniflora . Table S3: Site summary for relatedness for Spartina alterniflora . Table S4: Source summary for relatedness for Spartina patens . Table S5: Site summary for relatedness for Spartina patens . Table S6: Linear model summary for clone‐corrected S. alterniflora heterozygosity estimates among sites. Table S7: Linear model summary for S. patens heterozygosity estimates among sites. [file EVA-19-e70292-s001.docx]
